# Impact of sociodemographic, clinical, and intervention characteristics on pain intensity within a single music therapy session

**DOI:** 10.1016/j.jpain.2025.105556

**Published:** 2025-09-11

**Authors:** Samuel N. Rodgers-Melnick, Douglas Gunzler, Thomas E. Love, Siran M. Koroukian, Mark Beno, Jeffery A. Dusek, Johnie Rose

**Affiliations:** a Connor Whole Health, University Hospitals of Cleveland, Cleveland, OH, United States; b Department of Population and Quantitative Health Sciences, Case Western Reserve University School of Medicine, Cleveland, OH, United States; c Department of Psychiatry, Case Western Reserve University School of Medicine, Cleveland, OH, United States; d Population Health Research Institute, The MetroHealth System, Cleveland, OH, United States; e Department of Medicine, Case Western Reserve University School of Medicine, Cleveland, OH, United States; f Cleveland Institute for Computational Biology, Case Western Reserve University School of Medicine, Cleveland, OH, United States; g Department of Medicine, University of California – Irvine, Irvine, CA, United States; h Susan Samueli Integrative Health Institute, University of California – Irvine, Irvine, CA, United States; i Center for Community Health Integration, Case Western Reserve University School of Medicine, Cleveland, OH, United States

**Keywords:** Music therapy, Electronic health record, Pain, Guided imagery

## Abstract

Several studies support the efficacy of music therapy (MT) for reducing pain, but few have examined which interventions are most effective or which patients are more likely to respond. This study investigated which sociodemographic, clinical, and intervention characteristics are associated with clinically significant reductions in pain intensity (0–10 numeric rating scale reduction ≥2 units) within a single MT session. We conducted a retrospective review of 2039 MT sessions provided across a large health system among 1203 adult patients reporting pre-session pain ≥4/10, a complete post-session pain score, and a complete pre-session stress score. We employed a multivariable logistic mixed effects model to evaluate binary pain reduction response (≥2 units vs. < 2 units) where patients were considered nested within therapists. The model included a fixed covariate for MT intervention type: receptive only, recreative (i.e., singing or active instrument play), compositional/creative (e.g., songwriting), or music-assisted relaxation and imagery (MARI). Covariates in the model associated with higher adjusted odds ratios (aOR [95% CI]) included (1) recreative (1.37 [1.00, 1.86]) and MARI (1.48 [1.01, 2.17]) MT interventions as compared to receptive; (2) 15-minute increases in session length (1.40 [1.22, 1.61]); (3) 1-unit increases in pre-session pain (1.19 [1.11, 1.28]), (4) 5-unit increases in Elixhauser comorbidity count (1.29 [1.05, 1.60); and (5) a documented MT session goal of pain management (3.58, [2.64, 4.86]). MT interventions involving singing, active instrument play, and relaxation/imagery may be more effective for reducing pain intensity than interventions only involving live or recorded music among patients with high pre-session pain.

**Perspective:**

This study examined factors associated with meaningful reductions in pain (0–10 numeric rating scale reduction ≥2 units) within a single music therapy session. Among hospitalized patients, interventions involving singing, active instrument play, and relaxation/imagery may be more effective for reducing pain than interventions only involving live or recorded music.

## Introduction

Within every medical center, most patients will experience elevated acute pain at some point during their stay.^1^ If this pain is not well-managed, patients may experience significant psychological distress and persistent impairment following hospital discharge, which will ultimately slow their recovery.^2,3^ Therefore, healthcare professionals (HCPs) have a responsibility to address patients’ acute pain, but this is challenging given the risks and adverse events associated with opioids, one of the primary tools HCPs use to address acute pain.^3^ An estimated 50% of chronic opioid use begins within inpatient care,^4^ and there is a high probability of long-term opioid use after as little as five days of opioid treatment.^5^ Given the continued importance of inpatient pain management, the need to minimize opioid exposure, and the directives from accrediting bodies such as the Joint Commission to promote and provide nonpharmacologic pain treatments,^6,7^ many health systems are now making an intentional shift from relying on opioids toward providing evidence-based nonpharmacologic modalities.^2,8^

Music therapy (MT) is one such modality that has demonstrated efficacy for addressing acute pain. MT is the clinical use of tailored music interventions (e.g., active music making, music-assisted relaxation and imagery [MARI], and songwriting) to accomplish individualized goals within a therapeutic relationship by a credentialed professional (i.e., a board-certified music therapist [MT-BC]).^9^ This therapeutic relationship between the MT-BC and the patient makes MT distinct from other music-based interventions such as music medicine where HCPs such as nurses provide patients with recorded music interventions. Several randomized controlled trials (RCTs) support MT’s efficacy for addressing acute pain within populations including oncology,^10^ inpatient palliative care,^11^ and orthopedic surgery.^12^ A 2016 meta-analysis of 97 music-based intervention studies found that MT had a more clinically meaningful effect (−1.50, *p* <.001) on reducing numeric rating scale (NRS) measures of pain intensity than music medicine (−1.08, *p* <.001).^13^ Although the mechanisms by which music-based interventions affect pain are still being investigated, several cognitive^14,15^ and neurobiological mechanisms (e.g., modulating descending pain pathway)^16,17^ have been demonstrated in prior studies.

With MT now being integrated within health systems,^18^ recent observational studies have begun evaluating its real-world effects. Several studies support MT’s clinical effectiveness for reducing pain intensity among inpatient populations including hematology/oncology,^19–21^ palliative care,^22^ and individuals reporting moderate-to-severe pain (i.e., ≥4/10 on the 0–10 NRS) within community hospitals.^23^ However, there remain gaps in understanding which patient and MT intervention characteristics are associated with changes in pain intensity. Our prior analysis within community hospitals found that patients receiving an MT session in which the music therapist labeled “pain management” as their therapeutic goal were 4.32 times more likely (95% confidence interval [CI] 2.26, 8.66) to report pain reduction of ≥2 units than patients receiving an MT session in which pain management was not a session goal. However, this study did not examine the effects of specific MT intervention types or control for covariates such as opioid exposure and social drivers of health (SDoH).^23^ Accordingly, to fill this knowledge gap, the purpose of this study was to investigate which sociodemographic, clinical, and intervention characteristics are associated with clinically significant reductions in pain intensity (i.e., NRS reduction ≥2 units) within a single MT session among hospitalized patients. Given recent neuroscientific reports^24^ and our prior work among adults with sickle cell disease (SCD),^25,26^ we hypothesized that compared to purely receptive MT sessions, there would be higher odds of pain reduction ≥2 units within sessions incorporating active (e.g., instrument play, singing) and relaxation/imagery components after accounting for other socio-demographic and clinical characteristics.

## Methods

### Participants and design

This study is a retrospective electronic health record (EHR) review of individualized MT sessions provided to non-Hispanic (NH) White or NH Black/African American adult patients ≥18 years between August 03, 2020 and July 28, 2023. We limited the sample to individuals coded as NH White or NH Black/African American within the EHR because of limited prevalence (< 1.3%) of individuals from other racial and ethnic groups. To be included within the sample, patients had to report a pre-session NRS pain intensity score ≥4/10, a complete pre-session stress NRS score, and a complete post-session NRS pain intensity score within a single MT session. Sampling was not limited based on patients’ presenting diagnoses.

### Setting and care delivery

Music therapists provided MT sessions across ten medical centers within University Hospitals (UH), a non-profit health system in Northeast Ohio serving more than 1.2 million patients per year. MT services within the UH system are provided without cost to patients (i.e., not billed to insurance) and funded through multiple sources including foundation grants, philanthropy, and each hospital’s operating budget. Music therapists are integrated within clinical care and collaborate with other HCPs (e.g., physicians, advanced practice providers, nurses, social workers, chaplains, etc.) to address patients’ symptoms and enhance psychosocial support. Within the UH health system, various HCPs (e.g., physicians, nurses, advanced practice providers) place EHR referrals to address patients’ physical and psychosocial needs including coping, anxiety reduction, pain management, and mood modification, among others.^18^

Following an EHR referral from the medical team, MT-BCs and/or MT interns supervised by MT-BCs (1) conduct comprehensive assessments of patients’ symptoms (i.e., pain, stress, and anxiety rated on the 0–10 NRS), stressors, coping skills, and music preferences; (2) develop MT intervention plans to address patients’ specific needs in collaboration with patients and their families; (3) engage patients in one or more interventions such as active music making, songwriting, and/or MARI where selected music is tailored to patients’ preferences; (4) evaluate patients’ responses to treatment, including post-session symptom ratings on the NRS; and (5) document their sessions within a structured EHR documentation template. This EHR template was refined through a quality improvement initiative^27^ and served as the primary data source for this study. We trained all MT-BCs to use discrete free-text, checkbox, and radio button fields within this structured EHR template to document which MT intervention(s) they provided as well as patients’ reported pain, stress, and anxiety pre- and post-session.^27^

### Ethics and permissions

This study was approved by the UH Cleveland Medical Center Institutional Review Board as a retrospective chart review (STUDY20191213) with a waiver of informed consent.

### Data collected

We extracted all data from the UH Allscripts EHR and Electronic Data Warehouse (EDW) using multiple structured query language (SQL) scripts. These data included (1) socio-demographic information including age, sex, race/ethnicity, marital status, insurance status, and social vulnerability index (SVI)^28^; (2) clinical characteristics including Elixhauser comorbidity count, receipt of palliative care, and presence of key pain-related diagnoses based on International Classification of Diseases (ICD)-10 codes for SCD, neoplasms, and mental health/substance use disorders (MSUD)^29^; (3) whether or not the patient received opioids within 12 h prior to the MT session; (4) patients’ pre- and post-session ratings of pain, stress, and anxiety on the 0–10 NRS; and (5) MT intervention characteristics including intervention category, length (minutes), the music therapist’s years of experience, and the documented goals of the session.

We summarize the four main MT intervention categories used for analysis in Table 1. Within the structured EHR MT documentation template, music therapists could specify the intervention(s) they provided to patients. From August 2020 to March 2021, we instructed therapists to use specific terms within a free-text field to specify their MT interventions. We monitored these fields regularly to ensure consistent use.^27^ A revised documentation template was implemented in March 2021 and included a multi-select checkbox list rather than a free-text field to improve data consistency. We developed this pre-specified list after reviewing free-text descriptions of over 15,000 MT interventions and consolidating similar interventions under distinct intervention categories.^27^ These categories included (1) *live music listening,* therapist provides live music without imagery while the patient listens or discusses music; (2) *recorded music listening*, therapist provides recorded music while patient listens or discusses music; (3) *music listening not otherwise specified (NOS)*, therapist provides music not specified as live or recorded (in the former free-text documentation field) while patient listens or discusses music; (4) *active music making*, patient engages in making music on any instrument including voice; (5) *therapeutic music instruction*, therapist provides specific instruction on how to play a musical instrument; (6) *music* + *movement*, patient is engaged in directed body movements during music intervention; (7) *music-assisted relaxation and imagery (MARI)*, therapist engages patient with live/recorded music and guided relaxation of any kind (e.g., breathing, guided relaxation, autogenic relaxation, progressive muscle relaxation, imagery); (8) *songwriting*, therapist assists patient in creating a new song; (9) *song dedication*, patient creates a song dedicated to a person in their life; (10) *song recording*, patient records a song that they created; (11) *music-assisted life review*, therapist uses music to help patient reflect, reminisce, and/or re-examine the past; (12) *lyric analysis*, therapist engages patient in analyzing specific lyrics of a song; (13) *iso-principle*, therapist uses musical elements (e.g., tempo/dynamics) to match patient’s current state, and then shift musical elements in the desired direction to effect change.

We then consolidated these 13 intervention types into 4 distinct and mutually exclusive categories as follows: (1) *Receptive only*, where the music therapist engaged patients with live music listening, recorded music listening, music listening NOS, or lyric analysis, but the patient did not engage in any active music making (e.g., instrument play, singing), composition (i.e., songwriting, song dedication, music-assisted life review, or song recording), or relaxation/imagery techniques; (2) *Recreative*, where the patient engaged in some instrument play or singing (i.e., active music making, therapeutic music instruction, or music + movement) along with live or recorded music, but no composition techniques (i.e., songwriting, song dedication, music-assisted life review, or song recording); (3) *MARI* where the patient did not engage in instrument play, singing, or composition, but did engage in relaxation/imagery techniques along with live or recorded music; and (4) *Compositional/creative,* where the patient engaged in composition (i.e., songwriting, song dedication, music-assisted life review, or song recording), possibly in combination with instrument play, singing, or MARI techniques. These four distinct categories were developed based on definitions from Clements-Cortes^30^ and collaborative discussions with other MT-BCs and MT researchers. We coded MT interventions solely based on discrete data available from EHR documentation rather than individual observations of intervention recordings.

We calculated Elixhauser comorbidities using the “comorbidity” package.^31^ Total opioid exposure was calculated after curating data on oral, intravenous, patch, and patient-controlled analgesia exposures and converting to oral morphine equivalents using guidance from McPherson.^32^ The NRS is a validated and widely-used measure for acute pain intensity.^33^ NRS measures of stress and anxiety have also been widely used within observational studies of inpatient integrative health and medicine (IHM) modalities^23,34–37^ including MT.^21,23,27^

### Data analysis

The pain intensity outcome was modeled as a binary variable of (0) pain reduction < 2 units or (1) pain reduction ≥2 units, consistent with our prior research examining predictors of pain response within community hospitals^23^ and prior acupuncture^38^ and MT^39^ studies among patients with cancer where NRS reductions ≥2 units were defined as clinically significant. This binary classification was also better suited toward understanding associations with clinically meaningful pain responses than examining pain intensity as a continuous variable where small (e.g., 0.3 units) but statistically detectable associations would be less meaningful and harder to interpret. We chose model covariates based on their availability within the EHR, associations with pain in previous studies, and their role in examining predictors of pain intensity change in prior studies of inpatient IHM modalities.^23,40^ Specifically, pre-session patient-reported outcome measures were chosen as they have been shown to affect response to IHM modalities in prior studies.^41^ Selected socio-demographic variables were chosen given (1) increased chronic pain prevalence with age^42^; (2) multiple studies have described pain intensity and treatment response differences by sex^41,43^; (3) the history of racial bias among HCPs treating pain, racial disparities in pain management,^44^ and findings from a recent study where Black patients with cancer receiving MT reported higher pre-session pain (4.2 vs. 3.1 on the Edmonton Symptom Assessment Scale) than white patients with cancer receiving MT^20^; (4) the association between marital status and higher pain intensity among female participants undergoing cardiac surgery^45^; and (5) prior research demonstrating associations between neighborhood-level SDoH and higher pain intensity.^46–51^

We selected clinical covariates based on their demonstrated associations with pain intensity in prior studies. Our prior work examining inpatient MT and massage therapy revealed that SCD diagnosis (as compared to other hematology/oncology diagnoses) is associated with higher pre-session pain intensity among adults^21^ and pediatric patients.^52^ Prior studies have also shown that MSUD^53^ diagnoses and increases in Elixhauser comorbidity count^54^ are associated with higher pain intensity. We chose opioid receipt within 12 h prior to the MT session as a covariate to control for the effect of opioids on pain intensity within the MT session. Finally, we selected MT intervention characteristics to understand whether differences in patients’ engagement in MT, increases in session length, therapists’ years of experience (i.e., intern, MT-BC <5 years, or MT-BC ≥5 years based on distance from board-certification date), and documented pain management goal were associated with the outcome.

We examined all covariates for missingness prior to outcome analysis, with all missing data assumed to be missing at random (i.e., probability of missing data depended upon available information from other covariates). Of 2039 MT sessions, 303 (14.9%) had missing values, the most common being pre-session-anxiety (12.8% missing) followed by session length (0.83% missing), SVI (0.78% missing), insurance category (0.69% missing), and Elixhauser comorbidity count (0.05% missing). We performed single imputation procedures using decision tree and predictive mean matching from the “simputation” package.^55^ Descriptive statistics and bivariate analyses including Pearson’s Chi squared for categorical variables and Wilcoxon rank sum tests for continuous variables were conducted using the tbl_summary function from the “gtsummary” package.^56^

**Fig. 1.**
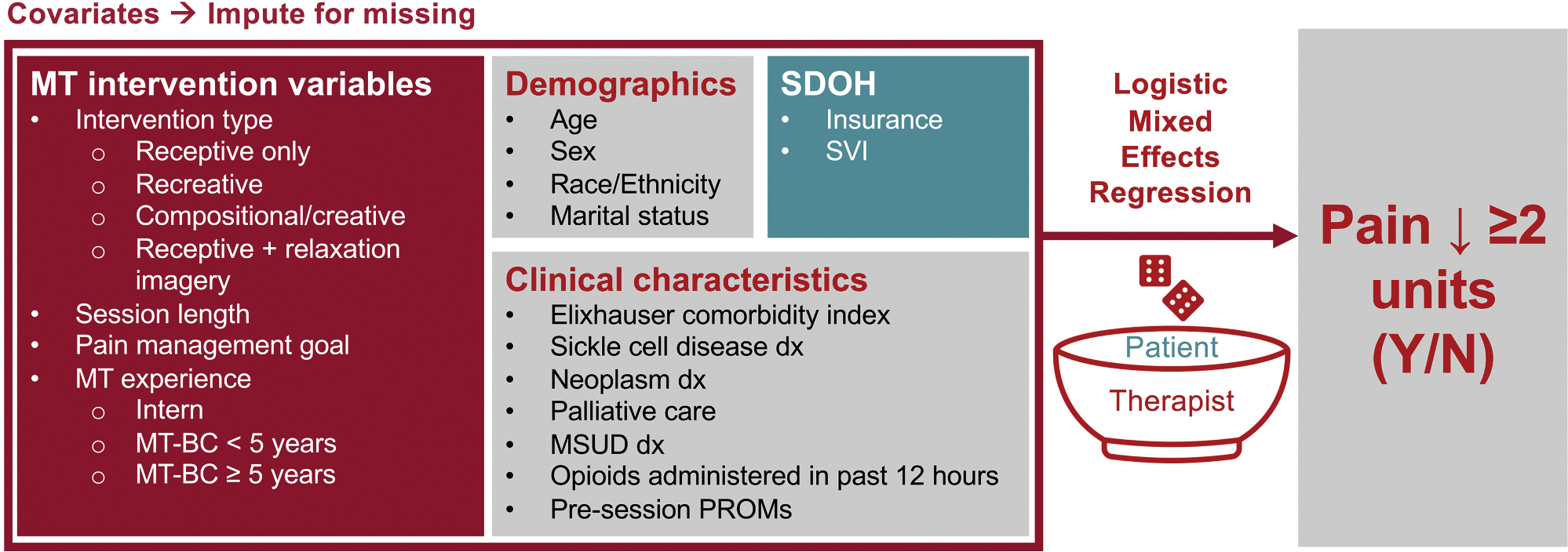
Schematic of Logistic Mixed Effects Regression Model. Covariate categories including music therapy intervention variables, demographics, social drivers of health, and clinical characteristics were used to predict the binary pain reduction response (≥2 units vs. < 2 units) within a multivariable logistic mixed effects model. Given that some patients appeared in multiple sessions within the sample, the model adjusted for the random effect (represented by the dice) of patients nested within therapists to control for individual patient- and therapist-level effects. Abbreviations: dx, diagnosis; MSUD, mental health or substance use disorder; MT, music therapy; MT-BC, music therapist-board certified; PROMs, patient-reported outcome measures; SDOH, social drivers of health; SVI, social vulnerability index; Y/N, yes/no.

We fit a logistic mixed effects regression model (Fig. 1) using the glmer function from the “lme4” package.^57^ Continuous variables were transformed to enable meaningful interpretations of coefficients (i.e., age in 10-year increments, scaled and centered SVI, Elixhauser comorbidity counts in 5-unit increments, and session length in 15-minute increments). Given that some patients appeared in multiple sessions within the sample, the model adjusted for the random effect of patients nested within therapists to control for individual patient- and therapist-level effects. Intraclass correlation coefficients (ICCs) were calculated to examine the level of clustering within the model. We examined model performance (e.g., posterior predictive check, collinearity, uniformity of residuals) using the check_model function from the “performance” package.^58^ All analyses and plots were generated using R Version 4.4.2^59^ and RStudio Version 2024.12.0+467.^60^

## Results

### Sample characteristics

The final sample included 2039 MT interventions (mean ± standard deviation length: 35.81 ± 14.51 min). These MT interventions detailed in Table 1 primarily addressed goals including, but not limited to pain management (64.2%), coping (41.0%), stress reduction (40.0%), anxiety reduction (35.5%), and relaxation (13.6%). Sessions were delivered by 29 different MT clinicians with years of clinical experience ranging from pre-internship completion (0 years) to 20.2 years (mean years of experience 3.66 ± 3.49 since board certification). Therapists provided sessions to 1203 unique patients (mean age 58.29 ± 17.20 years) who were predominantly female (66.2%) and coded as NH White (67.1%) or NH Black/African American (32.9%) within the EHR. Of these 1203 patients, 879 (73.1%) received just 1 session, and 324 (26.9%) received >1 session within the sample. MT sessions were delivered across 1484 distinct medical center encounters, including 737 (49.7%) academic and 747 (50.3%) community medical center encounters with a median (interquartile range) length of stay of 6 (3–12) days. Of all encounters, 961 (64.8%) included MSUD diagnoses, with the most common being depressive disorders (41.8%), anxiety disorders (40.7%), and trauma- and stressor-related disorders (11.7%). Patients presented to medical centers with various diagnoses (36.0% hematology/oncology including 24.7% neoplasm and 12.9% SCD) and a mean of 5.98 ± 3.29 Elixhauser comorbidities that included uncomplicated hypertension (58.8%), fluid and electrolyte disorders (50.5%), cardiac arrythmias (35.9%), chronic pulmonary disease (35.9%), and obesity (34.9%).

### Bivariate analysis

Table 2 presents the bivariate comparison of MT sessions. Compared to sessions in which pain was reduced < 2 units (*N* = 1131), sessions in which pain was reduced ≥2 units (*N* = 908) included older patients (mean 55.94 v. 52.67 years, *p*<.001), a larger proportion of female patients (65.5% v. 60.4%, *p*=.017), and a smaller proportion of single patients (41.0% v. 48.3%, *p*=.006) and patients receiving Medicaid (28.7% v. 33.8%, *p*=.044). These 908 sessions were delivered to patients from neighborhoods with lower SVI (mean 0.62 v. 0.65, *p*=.014) and patients with a greater number of Elixhauser comorbidities (6.37 v. 5.91, *p*=.006). SCD diagnosis was less prevalent within this group (10.5% v 16.1%, *p*<.001), while neoplasm diagnoses were more prevalent (27.6% v. 23.3%, *p*=.026). These sessions were also longer (37.63 v. 34.35 min, *p*<.001), documented with a pain management goal at a higher rate (75.2% v. 55.3%, *p*<.001), and delivered to patients rating higher pain intensity (7.25 v. 6.85, *p*<.001) and anxiety (4.97 v. 4.58, *p*=.013) than sessions in which patients reported pain reduction < 2 units. MT intervention category proportion varied between groups (*p*=.049). No meaningful between-group differences were observed in race/ethnicity, receipt of palliative care, MSUD diagnosis, opioid receipt, or music therapist experience.

### Logistic mixed effects model

**Fig. 2.**
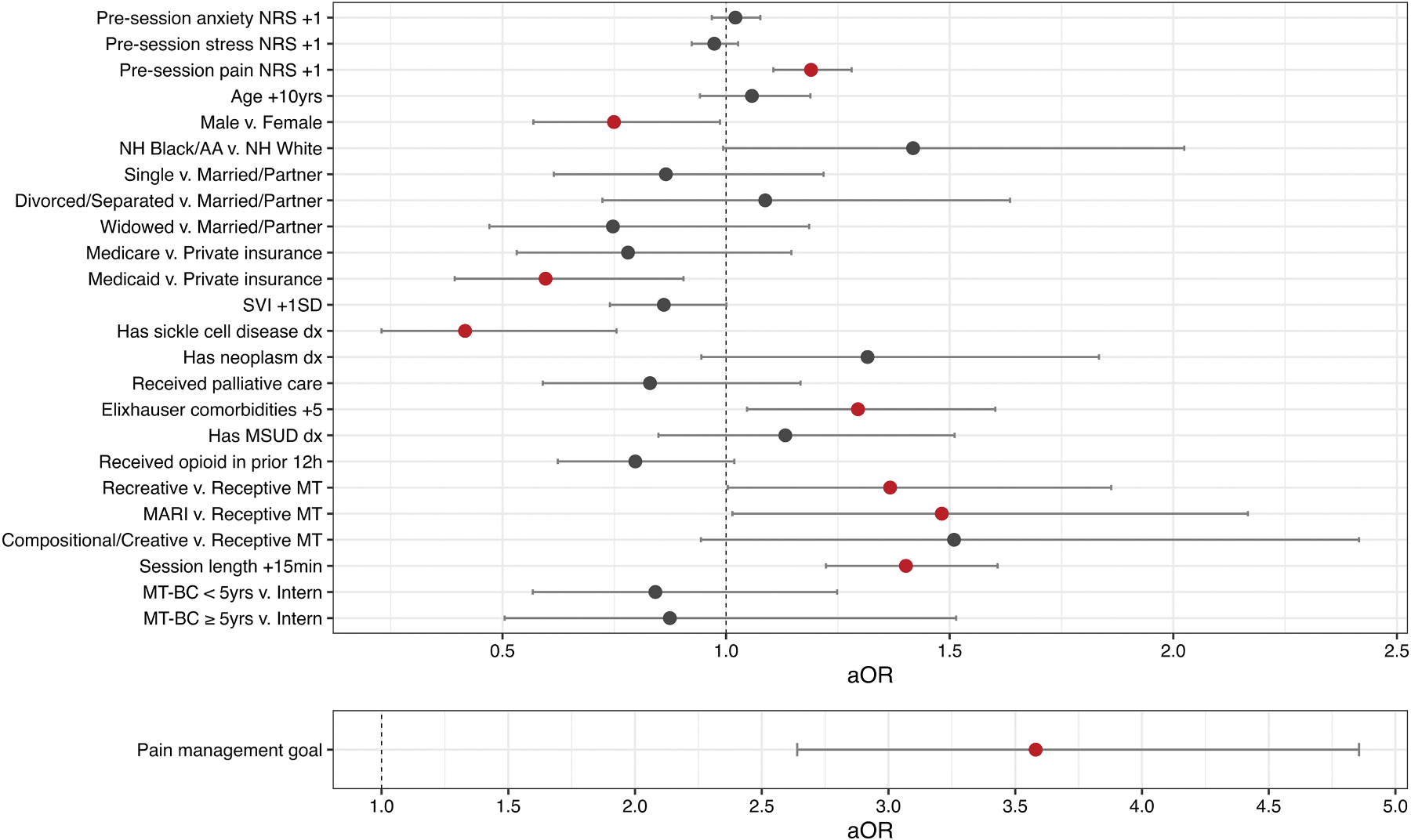
Adjusted Odds Ratios from the Logistic Mixed Effects Regression Model. Dots represent point estimates for adjusted odds ratios, and error bars represent 95% confidence intervals. Red dots are covariates where the confidence interval did not include 1. The pain management goal covariate is placed on a separate axis given its size to not obscure the other covariates. “_+”_ denotes the amount of increase in the continuous covariate (e.g., +1 NRS unit, +15min) associated with the plotted adjusted odds ratio. Abbreviations: dx, diagnosis; h, hours; MSUD, mental health/substance use disorder; MARI, music-assisted relaxation and imagery; min, minutes; MT, music therapy; MT-BC, music therapist-board certified; NRS, numeric rating scale; NH, non-Hispanic; SD, standard deviation; SVI, social vulnerability index; yrs, years.

Table 3 and Fig. 2 present adjusted odds ratios (aOR) and 95% CI from the logistic mixed effects model. The following were associated with increased odds (aOR, [95% CI]) of pain reduction ≥2 units after adjusting for all other covariates: (1) patients’ pre-session pain intensity ratings increasing 1 unit on the NRS (1.19 [1.11, 1.28]); (2) Elixhauser comorbidities count increases of 5 units (1.29 [1.05, 1.60]); (3) recreative (1.37 [1.00, 1.86], *p* =.047) and MARI (1.48 [1.01, 2.17]) MT interventions as compared to receptive; (4) session length increases of 15 min (1.40 [1.22, 1.61]); and (5) a documented pain management goal (3.58 [2.64, 4.86]). By contrast, the following were associated with decreased odds of pain reduction ≥2 units: (1) male as compared to female sex (0.75 [0.57, 0.99]); (2) having Medicaid as compared to private insurance (0.60 [0.39, 0.90]); and (3) having an SCD diagnosis (0.42 [0.23, 0.76]). The ICC for patients nested within therapists was .216, suggesting that there was variation across clusters and that the two-level nesting structure was appropriate within the model. Model assumptions were met with good performance in posterior predictive checks, uniformity of residuals, and normality of random effects.

## Discussion

This study examined which characteristics were associated with clinically significant reductions in pain intensity within a single MT session. Findings suggest that analgesic response may be influenced by certain socio-demographic characteristics, SCD diagnosis, comorbidities, session length, and MT intervention type. This study is the first to compare the effects of different MT interventions using real-world EHR data collected throughout a large health system. Our findings support the unique role MT-BCs provide in engaging patients in singing, instrument play, relaxation, and imagery within music interventions to manage pain. By tailoring interventions to patients’ preferences and abilities, engaging and maintaining patients’ focus on active interventions, and responding to patients’ needs in the moment, MT-BCs provide unique and essential services that go beyond receptive music listening.^25,61,62^ Given the observed effect of increased session length, the time that MT-BCs have to implement these services within medical centers also has a meaningful effect on analgesic response.

The observed effects of various sociodemographic, clinical, and intervention covariates may be explained by factors such as how particular populations perceive the NRS, the influence of stress and SDoH on pain, and the differences in how patients engaged with and responded to active versus receptive interventions. The finding that higher pre-session pain was associated with higher odds of pain reduction ≥2 units is consistent with studies of acupuncture among adults with chronic pain^41^ and veterans with persistently elevated NRS scores.^53^ Mathematically, individuals who rate their pain higher have a greater range within the NRS to respond (floor/ceiling effects). Similarly, the observed relationship between increased Elixhauser comorbidities and pain response is consistent with a cohort study of 12,924 veterans with persistently elevated NRS scores in which higher Selim comorbidity scores were associated with greater likelihood of pain improvement over 12 months (aOR ≥8 vs 0–3 comorbidities: 4.77 [2.19, 7.34]).^53^

Prior studies have demonstrated similar sex-based differences in analgesic response. In a large (*N*=9990) analysis of 4 German RCTs among patients with chronic low back pain, headache, neck pain, or osteoarthritis,^41^ female sex was associated with higher odds of having a 5-unit improvement on the SF-36 pain subscale. An EHR review of 11, 000 patients who had pain scores recorded as part of routine care uncovered significantly higher self-reported pain in women compared with men across 14 different diagnoses.^63^ Thus, the observed differences in pain response may be related to higher pre-session pain. Historically, women have also been more likely to engage in^64^ and favor IHM modalities^41^ as well as engage in emotion-focused tactics, attentional focus, cognitive reframing, and social support techniques for pain management that share similarities with MT.^43,65,66^ Given the findings of the current study, future studies should consider using mixed-methods clinical trial approaches to further explore differences in analgesic response based on sex and gender.

Our finding that patients with SCD were less likely to report pain reduction ≥2 units is significant given the severity of vaso-occlusive crises^67^ and prior studies supporting MT’s benefits for acute pain in this population.^25^ The SCD pain experience is unique given (1) the positive association between racism-based discrimination in healthcare settings and increased depressive symptoms, insomnia, and daily pain severity^68^; (2) the high frequency of pain crises throughout the lifespan^69^; and (3) the early development of central sensitization^70^ and other chronic pain syndromes within this population. Our prior work demonstrated that individuals with SCD rate their pre-session pain higher (7.22 v. 5.81) than those with other hematologic/oncologic conditions participating in MT.^21^ However, the NRS has significant limitations within this population. A qualitative study conducted with 48 youth with SCD found that the NRS failed to evaluate relational aspects of pain and contributed to misunderstanding and mistrust of the medical system.^71^ In this study, some patients reported a fear that reporting a lower pain score would hasten discharge or delay their next dose of pain medications, while others described how their pain intensity in a given moment may not reflect their expected pain later in a crisis.^71^ Thus, our findings may be explained by the unique ways in which patients with SCD perceive their pain intensity rather than a fundamental propensity to respond less to MT.

Medicaid beneficiaries were less likely to report pain reduction ≥2 units than those with private insurance (aOR 0.60 [0.39, 0.90]). Also, while the CI did include 1, higher SVI was also associated with decreased odds of reporting this response (0.86 [0.74, 1.00], *p* = 0.051). These findings may be explained by known associations between neighborhood disadvantage, stress, and pain intensity.^46–51^ A cross-sectional study of 673 racially-diverse adults (38% Black, 38% Latino, 24% Asian) with self-reported chronic pain found a positive relationship between stressful life events and pain intensity (β =.20, *p*<.001).^72^ Increased perceived stress is associated with increased pain intensity,^73, 74^ and prolonged exposure to stress can alter neurobiological pathways (e.g., cortisol secretion, upregulation of pro-inflammatory genes) through which the brain processes pain.^46,48,75^ Therefore, MT-BCs working with patients in moderate-to-severe pain should consider the influence of patients’ stressors and SDoH on analgesic response.

MT interventions in which patients engaged in instrument play and/or singing were more likely to result in clinically meaningful pain reduction than receptive interventions (aOR 1.37 [1.00, 1.86], *p*=.047). This finding is similar to our prior work among adults with SCD in which a 20-minute electronic music improvisation session with an MT-BC was associated with pain intensity reduction ≥1.25 units (5.12, *p*=.025), while receptive listening to recorded music did not have the same strength of association (3.63, *p*=.096). Another study among 236 patients with cancer reporting moderate-to-severe fatigue found that active MT interventions (i.e., singing, instrument playing, writing, and/or movement) were associated with a 0.88-unit greater reduction in fatigue than receptive MT interventions.^76^

From a mechanistic perspective, recreative interventions involve sensorimotor synchronization (e.g., playing an instrument), which may enhance analgesic response.^24^ A recent study among 59 adults exposed to an experimental pain stimulus found that tapping with music (but not simply listening to music) yielded large effects on reducing pain (*d*=0.93), while tapping without music did not elicit such an effect.^77^ Similarly, in an RCT conducted among 286 adults reporting pain intensity ≥2/10 on the NRS, participants reporting higher levels of active musical engagement experienced greater decreases in pain intensity as compared to those who reported lower levels of active engagement.^78^ Compared to receptive interventions, recreative methods may also enhance cognitive processes (e.g., refocused attention, motivation, self-efficacy, meaning, and enjoyment) that contribute to analgesic response.^14,15^

Results from this study also support the role of guided relaxation, breathwork, and imagery within MT interventions. MARI interventions have demonstrated benefits for reducing pain intensity (difference in means −1.39 [−1.95, −0.83] *p*<.001) among patients receiving inpatient palliative care^11^ and reducing pain interference (−2.10 ± 4.68, *p*=0.016) among patients with SCD.^79^ Imagery, whether visual or imagined, can intensify patients’ emotional response to music.^80^ From the perspective of Melzack’s Neuromatrix Theory,^81^ MT-BCs facilitating MARI interventions may specifically modulate the sensory-descriptive dimension of pain through inviting patients to imagine sights, sounds, smells, and textures associated with a peaceful place, take deeper diaphragmatic breaths, or release tension from the body.^82^

Strengths of this study include (1) a large sample size consisting of diverse clinical populations across 10 medical centers where health insurance was not a barrier to receiving MT services; (2) high Black/African American representation as compared to other inpatient IHM studies^8^; (3) a novel approach to using EHR data to measure MT’s real-world effectiveness; (4) collection of NRS measures immediately before and after MT sessions among patients with moderate-to-severe symptoms; and (5) including opioid exposure and SDoH as covariates. Important limitations include the use of single-item NRS measures rather than more comprehensive pain instruments and a broad categorization of MT interventions which may not have captured nuanced aspects of treatment (e.g., therapists’ theoretical orientations, patients’ preferences, and use of specific instruments, tempos, or songwriting techniques). The present analysis is limited to single-session effects rather than longitudinal changes in pain intensity over the course of a hospital admission. This sample of patients referred to MT may not be representative of all U.S. inpatients, as MSUD were far more prevalent (64.8%) than prior national estimates (27.8%).^29^ Additionally, as with many retrospective EHR studies, demographic data were extracted exactly as they were entered into the EHR by HCPs and may not have reflected the gender, racial, and/or ethnic identities of the patients included in this study.^83^

## Conclusion

Results of this study suggest that MT interventions involving singing or active instrument play and those involving guided relaxation, imagery, or breathing exercises may be more effective for reducing pain intensity than interventions only involving live or recorded music, especially when MT sessions are longer. Male patients, Medicaid beneficiaries, and patients with SCD may be less likely to report pain reduction ≥2 units following a single MT intervention, while patients reporting higher pain intensity and patients with more comorbidities may be more likely. Additional research is needed to confirm the comparative effects of MT interventions within RCTs, understand longitudinal effects on pain beyond a single session, and evaluate the mechanisms underlying differences in pain response.

## Figures and Tables

**Table 1:** Music therapy interventions.

Intervention	Receptive only N = 1050	Recreative N = 533	MARI N = 255	Compositional/creative N = 201

Live music listening	889 (84.7%)	168 (31.5%)	73 (28.6%)	50 (24.9%)
Recorded music listening	62 (5.9%)	42 (7.9%)	3 (1.2%)	14 (7.0%)
Music listening NOS	140 (13.3%)	0 (0.0%)	0 (0.0%)	1 (0.5%)
Active music making	0 (0.0%)	517 (97.0%)	0 (0.0%)	64 (31.8%)
Therapeutic music instruction	0 (0.0%)	17 (3.2%)	0 (0.0%)	0 (0.0%)
Music + movement	0 (0.0%)	11 (2.1%)	0 (0.0%)	2 (1.0%)
MARI	0 (0.0%)	25 (4.7%)	255 (100.0%)	10 (5.0%)
Songwriting	0 (0.0%)	0 (0.0%)	0 (0.0%)	154 (76.6%)
Song dedication	0 (0.0%)	0 (0.0%)	0 (0.0%)	23 (11.4%)
Song recording	0 (0.0%)	0 (0.0%)	0 (0.0%)	18 (9.0%)
Music-assisted life review	0 (0.0%)	0 (0.0%)	0 (0.0%)	13 (6.5%)
Lyric analysis	18 (1.7%)	4 (0.8%)	4 (1.6%)	4 (2.0%)
Iso-principle	14 (1.3%)	2 (0.4%)	9 (3.5%)	5 (2.5%)

Abbreviations: MARI, music-assisted relaxation and imagery; NOS, not other-wise specified.

**Table 2:** Bivariate analysis.

Variable	Pain reduced < 2 units N = 1131	Pain reduced ≥ 2 units N = 908	*p*-value

**Age**	52.67 ± 18.25	55.94 ± 17.54	<0.001^a^
**Sex**			0.017^b^
Female	683 (60.4%)	595 (65.5%)	
Male	448 (39.6%)	313 (34.5%)	
**Race/Ethnicity**			0.126^b^
NH White	666 (58.9%)	565 (62.2%)	
NH Black/African American	465 (41.1%)	343 (37.8%)	
**Marital status**			0.006^b^
Married/Life partner	324 (28.6%)	276 (30.4%)	
Single	546 (48.3%)	372 (41.0%)	
Divorced/Separated	147 (13.0%)	145 (16.0%)	
Widowed	114 (10.1%)	115 (12.7%)	
**Insurance category**			0.044^b^
Private/Military	181 (16.0%)	165 (18.2%)	
Medicare	568 (50.2%)	482 (53.1%)	
Medicaid	382 (33.8%)	261 (28.7%)	
**SVI**	0.65 ± 0.28	0.62 ± 0.29	0.014^a^
**Elixhauser comorbidity count**	5.91 ± 3.26	6.37 ± 3.49	0.006^a^
**Has sickle cell disease**	182 (16.1%)	95 (10.5%)	<0.001^b^
**Has neoplasm dx**	264 (23.3%)	251 (27.6%)	0.026^b^
**Received palliative care**	301 (26.6%)	219 (24.1%)	0.199^b^
**Has MSUD**	767 (67.8%)	605 (66.6%)	0.570^b^
**Received opioid in prior 12hr**	694 (61.4%)	529 (58.3%)	0.155^b^
**Pre-session pain NRS**	6.85 ± 1.83	7.25 ± 1.80	<0.001^a^
**Pre-session anxiety NRS**	4.58 ± 3.42	4.97 ± 3.43	0.013^a^
**Pre-session stress NRS**	5.11 ± 3.49	5.37 ± 3.47	0.101^a^
**Intervention category**			0.049^b^
Receptive only	604 (53.4%)	446 (49.1%)	
Recreative	293 (25.9%)	240 (26.4%)	
MARI	122 (10.8%)	133 (14.6%)	
Compositional/creative	112 (9.9%)	89 (9.8%)	
**Session length (minutes)**	34.35 ± 13.83	37.63 ± 15.12	<0.001^a^
**Music therapist experience**			0.124^b^
Intern	236 (20.9%)	209 (23.0%)	
MT-BC <5yrs	463 (40.9%)	332 (36.6%)	
MT-BC ≥5yrs	432 (38.2%)	367 (40.4%)	
**Pain management session goal**	626 (55.3%)	683 (75.2%)	<0.001^b^

Abbreviations: dx, diagnosis; MARI, music-assisted relaxation and imagery; MSUD, mental health/substance use disorder; MT-BC, music therapist-board certified; NH, non-Hispanic; NRS, numeric rating scale; SVI, social vulnerability index; yrs, years

a Wilcoxon rank sum test

b Pearson’s Chi-squared test

**Table 3:** Adjusted odds ratios from logistic mixed effects model.

Fixed effect	aOR	95% CI	p-value

Pre-session anxiety +1 NRS unit	1.02	0.97-1.08	0.446
Pre-session stress +1 NRS unit	0.97	0.92-1.03	0.327
Pre-session pain +1 NRS unit	1.19	1.11-1.28	<0.001
Age +10yrs	1.06	0.94-1.19	0.345
Male v. Female	0.75	0.57-0.99	0.040
NH Black/African American v. NH White	1.42	0.99-2.02	0.054
Single v. Married/Partner	0.87	0.61-1.22	0.407
Divorced/Separated v. Married/Partner	1.09	0.72-1.63	0.686
Widowed v. Married/Partner	0.75	0.47-1.19	0.216
Medicare v. Private insurance	0.78	0.53-1.15	0.206
Medicaid v. Private insurance	0.60	0.39-0.90	0.015
SVI +1SD	0.86	0.74-1.00	0.051
Has sickle cell disease dx	0.42	0.23-0.76	0.004
Has neoplasm dx	1.32	0.94-1.83	0.104
Received palliative care	0.83	0.59-1.17	0.283
Elixhauser comorbidities +5	1.29	1.05-1.60	0.017
Has MSUD dx	1.13	0.85-1.51	0.398
Received opioid in prior 12 h	0.80	0.62-1.02	0.070
Recreative v. Receptive MT	1.37	1.00-1.86	0.047
MARI v. Receptive MT	1.48	1.01-2.17	0.042
Compositional/Creative v. Receptive MT	1.51	0.94-2.42	0.086
Session length +15min	1.40	1.22-1.61	<0.001
MT-BC < 5yrs v. Intern	0.84	0.57-1.25	0.392
MT-BC ≥ 5yrs v. Intern	0.87	0.50-1.51	0.632
Pain management goal	3.58	2.64-4.86	<0.001

Abbreviations: aOR, adjusted odds ratio; CI, confidence interval; dx, diagnosis; h, hours; MSUD, mental health/substance use disorder; MARI, music-assisted relaxation and imagery; min, minutes; MT, music therapy; MT-BC, music therapist-board certified; NH, non-Hispanic; SD, standard deviation; SVI, social vulnerability index; yrs, years. “+” denotes the amount of increase in the continuous covariate (e.g., +1 NRS unit, +15min) associated with the adjusted odds ratio.

## Data Availability

The datasets generated and/or analyzed during the current study are not publicly available due to privacy restrictions as the databases contain information that could compromise the privacy of research participants. However, the de-identified datasets are available from the corresponding author on reasonable request.

## References

[1] Salamon KS, Russell C, DeVinney D, Soprano CM. Quality improvement protocol: improving the use of nonpharmacological pain management strategies within the inpatient hospital setting. J Clin Med. 2024;13(6):1680. 10.3390/jcm13061680. 38541903 PMC10970717

[2] Tick H, Nielsen A, Pelletier KR, Evidence-based nonpharmacologic strategies for comprehensive pain care: the consortium pain task force white paper. Explore. 2018;14(3):177–211. 10.1016/j.explore.2018.02.001. 29735382

[3] Hyland SJ, Wetshtein AM, Grable SJ, Jackson MP. Acute pain management pearls: a focused review for the hospital clinician. Healthc. 2023;11(1):34. 10.3390/healthcare11010034 PMC9818465 36611494

[4] Callinan CE, Neuman MD, Lacy KE, Gabison C, Ashburn MA. The initiation of chronic opioids: a survey of chronic pain patients. J Pain. 2017;18(4):360–365. 10.1016/j.jpain.2016.11.001. 27919771

[5] Shah A, Hayes CJ, Martin BC. Characteristics of initial prescription episodes and likelihood of long-term opioid use - United States, 2006–2015. MMWR Morb Mortal Wkly Rep. 2017;66(10):265–269. 10.15585/mmwr.mm6610a1. 28301454 PMC5657867

[6] The Joint Commission. Pain Assessment and Management – Understanding the Requirements. Published July 25, 2023. Accessed March 22, 2024. https://www.jointcommission.org/standards/standard-faqs/hospital-and-hospital-clinics/leadership-ld/000002161/.

[7] The Joint Commission. Non-pharmacologic and non-opioid solutions for pain management. Published September 20, 2018. Accessed March 18, 2019. https://www.jointcommission.org/en-us/knowledge-library/newsletters/quick-safety/issue-44.

[8] Dusek JA, Griffin KH, Finch MD, Rivard RL, Watson D. Cost savings from reducing pain through the delivery of integrative medicine program to hospitalized patients. J Alter Complement Med. 2018;24(6):557–563. 10.1089/acm.2017.0203. PMC6006422 29474095

[9] American Music Therapy Association. What is Music Therapy? | What is Music Therapy? | American Music Therapy Association (AMTA). Published January 16, 2021. Accessed March 8, 2021. https://www.musictherapy.org/about/musictherapy/.

[10] Bradt J, Dileo C, Myers-Coffman K, Biondo J. Music interventions for improving psychological and physical outcomes in people with cancer. Cochrane Database Syst Rev. 2021;10(10):CD006911. 10.1002/14651858.CD006911.pub4. 34637527 PMC8510511

[11] Gutgsell KJ, Schluchter M, Margevicius S, Music therapy reduces pain in palliative care patients: a randomized controlled trial. J Pain Symptom Manag. 2013;45(5):822–831. 10.1016/j.jpainsymman.2012.05.008. 23017609

[12] Gallagher LM, Gardner V, Bates D, Impact of music therapy on hospitalized patients post-elective orthopaedic surgery: a randomized controlled trial. Orthop Nurs. 2018;37(2):124–133. 10.1097/NOR.0000000000000432. 29570546

[13] Lee JH. The effects of music on pain: a meta-analysis. J Music Ther. 2016;53(4):430–477. 10.1093/jmt/thw012. 27760797

[14] Howlin C, Rooney B. The cognitive mechanisms in music listening interventions for pain: a scoping review. J Music Ther. 2020;57(2):127–167. 10.1093/jmt/thaa003. 32249313

[15] Bradt J, Leader A, Worster B, Music therapy for pain management for people with advanced cancer: a randomized controlled trial. Psychooncology. 2024;33(10), e70005. 10.1002/pon.70005. 39450934 PMC11778920

[16] Sihvonen AJ, Pitkäniemi A, Särkämö T, Soinila S. Isn’t there room for music in chronic pain management? J Pain. 2022;23(7):1143–1150. 10.1016/j.jpain.2022.01.003. 35124251

[17] Dobek CE, Beynon ME, Bosma RL, Stroman PW. Music modulation of pain perception and pain-related activity in the brain, brain stem, and spinal cord: a functional magnetic resonance imaging study. J Pain 2014;15(10):1057–1068. 10.1016/j.jpain.2014.07.006. 25077425

[18] Rodgers-Melnick SN, Rivard RL, Block S, Dusek JA. Effectiveness of medical music therapy practice: integrative research using the electronic health record: rationale, design, and population characteristics. J Integr Complement Med. 2024;30(1):57–65. 10.1089/jicm.2022.0701. 37433198 PMC10795501

[19] Lopez G, Christie AJ, Powers-James C, The effects of inpatient music therapy on self-reported symptoms at an academic cancer center: a preliminary report. Support Care Cancer. 2019;27(11):4207–4212. 10.1007/s00520-019-04713-4. 30825024

[20] Lichtl A, Casaw C, Edwards J, Music therapy for pain in black and white cancer patients: a retrospective study. J Pain Symptom Manag. 2022. 10.1016/j.jpainsymman.2022.07.007. PMC9588734 35870654

[21] Rodgers-Melnick SN, Rivard RL, Block S, Dusek JA. Clinical delivery and effectiveness of music therapy in hematology and oncology: an EMMPIRE retrospective study, 15347354221142538 Integr Cancer Ther. 2022;21(21). 10.1177/15347354221142538. PMC9751180 36510393

[22] Gallagher LM, Lagman R, Rybicki L. Outcomes of music therapy interventions on symptom management in palliative medicine patients. Am J Hosp Palliat Care. 2018;35(2):250–257. 10.1177/1049909117696723. 28274132

[23] Rodgers-Melnick SN, Rivard RL, Block S, Dusek JA. Effectiveness of music therapy within community hospitals: an EMMPIRE retrospective study. PAIN Rep. 2023;8(3), e1074. 10.1097/pr9.0000000000001074. 37731473 PMC10508459

[24] Koelsch S, Bradt J. A neuroscientific perspective on pain-reducing effects of music: Implications for music therapy and mental well-being. Ann N Y Acad Sci. 2025. 10.1111/nyas.70015. 40729759

[25] Rodgers-Melnick SN, Matthie N, Jenerette C, The effects of a single electronic music improvisation session on the pain of adults with sickle cell disease: a mixed methods pilot study. J Music Ther. 2018;55(2):156–185. 10.1093/jmt/thy004. 29796596

[26] Rodgers-Melnick SN, Gam K, Debanne S, Little JA. Music use in adult patients with sickle cell disease: a pilot survey study. Music Ther Perspect. 2021;39(1):34–41. 10.1093/mtp/miaa026.

[27] Rodgers-Melnick SN, Block S, Rivard RL, Dusek JA. Optimizing patient-reported outcome collection and documentation in medical music therapy: process-improvement study. JMIR Hum Factors. 2023;10, e46528. 10.2196/46528. 37498646 PMC10415937

[28] Spielman SE, Tuccillo J, Folch DC, Evaluating social vulnerability indicators: criteria and their application to the Social Vulnerability Index. Nat Hazards. 2020;100(1):417–436. 10.1007/s11069-019-03820-z.

[29] Owens PL, Fingar KR, McDermott KW, Muhuri PK, Heslin KC Inpatient Stays Involving Mental and Substance Use Disorders, 2016. Published March 26, 2019. Accessed June 8, 2021. https://hcup-us.ahrq.gov/reports/statbriefs/sb249-Mental-Substance-Use-Disorder-Hospital-Stays-2016.pdf. 31063293

[30] Development Clements-Cortes A. and efficacy of music therapy techniques within palliative care. Complement Ther Clin Pr. 2016;23:125–129. 10.1016/j.ctcp.2015.04.004. 25986297

[31] Gasparini A comorbidity: an R package for computing comorbidity scores. J Open Source Softw. 2018;3(23):648. 10.21105/joss.00648.

[32] McPherson MLM. Demystifying Opioid Conversion Calculations: A Guide for Effective Dosing. 2nd Edition. American Society of Health-System Pharmacists; 2018.

[33] Paice JA, Cohen FL. Validity of a verbally administered numeric rating scale to measure cancer pain intensity. Cancer Nurs. 1997;20(2):88–93. 10.1097/00002820-199704000-00002. 9145556

[34] Johnson JR, Crespin DJ, Griffin KH, Finch MD, Dusek JA. Effects of integrative medicine on pain and anxiety among oncology inpatients. J Natl Cancer Inst Monogr. 2014;2014(50):330–337. 10.1093/jncimonographs/lgu030. 25749600 PMC4411536

[35] Johnson JR, Crespin DJ, Griffin KH, The effectiveness of integrative medicine interventions on pain and anxiety in cardiovascular inpatients: a practice-based research evaluation, 486–486 BMC Complement Alter Med. 2014;14(1). 10.1186/1472-6882-14-486. PMC4301797 25494710

[36] Johnson JR, Rivard RL, Griffin KH, The effectiveness of nurse-delivered aromatherapy in an acute care setting. Complement Ther Med. 2016;25:164–169. 10.1016/j.ctim.2016.03.006. 27062964 PMC12915488

[37] Rodgers-Melnick SN, Srinivasan R, Rivard RL, Adan F, Dusek JA. Immediate effects of integrative health and medicine modalities among outpatients with moderate-to-severe symptoms, 27536130241254070 Glob Adv Integr Med Health. 2024;13. 10.1177/27536130241254070. PMC11088302 38737216

[38] Miller KR, Patel JN, Symanowski JT, Edelen CA, Walsh D. Acupuncture for cancer pain and symptom management in a palliative medicine clinic. Am J Hosp Palliat Care. 2019;36(4):326–332. 10.1177/1049909118804464. 30286611

[39] Bates D, Rybicki L. The effects of music therapy in liquid and solid tumor oncology patients. e68-e68 J Pain Symptom Manag. 2016;52(6). 10.1016/j.jpainsymman.2016.10.133.

[40] Dusek JA, Rivard RL, Griffin KH, Finch MD. Significant pain reduction in hospitalized patients receiving integrative medicine interventions by clinical population and accounting for pain medication. J Alter Complement Med. 2021;27(S1):S28–S36. 10.1089/acm.2021.0051. PMC8035926 33788611

[41] Witt CM, Schützler L, Lüdtke R, Wegscheider K, Willich SN. Patient characteristics and variation in treatment outcomes: which patients benefit most from acupuncture for chronic pain? Clin J Pain. 2011;27(6):550–555. 10.1097/AJP.0b013e31820dfbf5. 21317771

[42] Zelaya CE, Dahlhamer JM, Lucas JW, Connor EM Chronic Pain and High-impact Chronic Pain Among U.S. Adults, 2019. National Center for Health Statistics. Published November 4, 2020. Accessed September 5, 2021. https://www.cdc.gov/nchs/products/databriefs/db390.htm.

[43] Osborne NR, Davis KD Chapter Eight - Sex and gender differences in pain. In: Moro E, Arabia G, Tartaglia MC, Ferretti MT, eds. Int Rev Neurobiol. Academic Press; 2022:277–307. 10.1016/bs.irn.2022.06.013 36038207

[44] Essien UR, Ifidon A, Sue KL. Black pain matters: prioritizing antiracism and equity in the opioid epidemic. J Hosp Med. 2021;16(10):638–639. 10.12788/jhm.3703. 34613902 PMC8494278

[45] Bjornnes AK, Lie I, Parry M, Association between self-perceived pain sensitivity and pain intensity after cardiac surgery. J Pain Res. 2018;11:1425–1432. 10.2147/JPR.S167524. 30122973 PMC6078187

[46] Kapos FP, Craig KD, Anderson SR, Social determinants and consequences of pain: toward multilevel, intersectional, and life course perspectives. J Pain. 2024;25(10), 104608. 10.1016/j.jpain.2024.104608. 38897311 PMC11402600

[47] Raasveld FV, Lans J, Valerio IL, Eberlin KR. Social deprivation is associated with increased pain in patients presenting with neuropathic pain. Plast Reconstr Surg Glob Open. 2024;12(6). 10.1097/GOX.0000000000005931. PMC11326464 39148658

[48] Overstreet DS, Pester BD, Wilson JM, Flowers KM, Kline NK, Meints SM. The experience of BIPOC living with chronic pain in the USA: biopsychosocial factors that underlie racial disparities in pain outcomes, comorbidities, inequities, and barriers to treatment. Curr Pain Headache Rep. 2023;27(1):1–10. 10.1007/s11916-022-01098-8. 36527589 PMC10683048

[49] Persaud Y, Okhomina V, Heitzer AM, The impact of socio-economic determinants of health on PedsQL and pain outcomes among individuals with sickle cell disease. Blood. 2023;142:250. 10.1182/blood-2023-188145.

[50] Choi HY, Graetz I, Shaban-Nejad A, Social disparities of pain and pain intensity among women diagnosed with early stage breast cancer. Front Oncol. 2022;12, 759272. 10.3389/fonc.2022.759272. 35211396 PMC8861323

[51] Khalatbari-Soltani S, Blyth FM. Socioeconomic position and pain: a topical review. Pain. 2022;163(10):1855–1861. 10.1097/j.pain.0000000000002634. 35297800

[52] Rodgers-Melnick SN, Bartolovich M, Desai NJ, Massage therapy for children, adolescents, and young adults: clinical delivery and effectiveness in hematology and oncology. Pedia Blood Cancer. 2023;70(4), e30243. 10.1002/pbc.30243. 36726036

[53] Dobscha SK, Lovejoy TI, Morasco BJ, Predictors of improvements in pain intensity in a national cohort of older veterans with chronic pain. J Pain. 2016;17(7):824–835. 10.1016/j.jpain.2016.03.006. 27058162 PMC4925248

[54] Axon DR, Eckert B. Association of number of comorbid conditions and pain among united states adults. Diseases. 2024;12(7):147. 39057118 10.3390/diseases12070147 PMC11276597

[55] van der Loo M Simputation Simple Imput R Package Version 028. 2022.

[56] Sjoberg DD, Whiting K, Curry M, Lavery JA, Larmarange J. Reproducible summary tables with the gtsummary package. R J. 2021;13(1):570–580. 10.32614/rj-2021-053.

[57] Bates D, Mächler M, Bolker B, Walker S. Fitting linear mixed-effects models using lme4. J Stat Softw. 2015;67(1). 10.18637/jss.v067.i01.

[58] Lüdecke D, Ben-Shachar MS, Patil I, Waggoner P, Makowski D. Performance: an R package for assessment, comparison and testing of statistical models. J Open Source Softw. 2021;6(60). 10.21105/joss.03139.

[59] R Core Team. R: A language and environment for statistical computing. Vienna, Austria: R Foundation for Statistical Computing; 2022.

[60] R Studio Team. RStudio: Integrated development for R. Boston, MA: RStudio, PBC; 2020.

[61] Loewy J Underlying music mechanisms influencing the neurology of pain: an integrative model. Brain Sci. 2022;12(10):1317. 10.3390/brainsci12101317. 36291251 PMC9599384

[62] Bradt J, Potvin N, Kesslick A, The impact of music therapy versus music medicine on psychological outcomes and pain in cancer patients: a mixed methods study. Support Care Cancer. 2015;23(5):1261–1271. 10.1007/s00520-014-2478-7. 25322972

[63] Ruau D, Liu LY, Clark JD, Angst MS, Butte AJ. Sex differences in reported pain across 11,000 patients captured in electronic medical records. J Pain. 2012;13(3):228–234. 10.1016/j.jpain.2011.11.002. 22245360 PMC3293998

[64] Rodgers-Melnick SN, Trager RJ, Love TE, Dusek JA. Engagement in integrative and nonpharmacologic pain management modalities among adults with chronic pain: analysis of the 2019 National health interview survey. J Pain Res. 2024;17:253–264. 10.2147/JPR.S439682. 38260001 PMC10800282

[65] Fillingim RB, King CD, Ribeiro-Dasilva MC, Rahim-Williams B, Riley JL 3rd. Sex, gender, and pain: a review of recent clinical and experimental findings. J Pain. 2009;10(5):447–485. 10.1016/j.jpain.2008.12.001. 19411059 PMC2677686

[66] Racine M, Tousignant-Laflamme Y, Kloda LA, Dion D, Dupuis G, Choiniere M. A systematic literature review of 10 years of research on sex/gender and pain perception - part 2: do biopsychosocial factors alter pain sensitivity differently in women and men? Pain. 2012;153(3):619–635. 10.1016/j.pain.2011.11.026. 22236999

[67] Darbari DS, Sheehan VA, Ballas SK. The vaso-occlusive pain crisis in sickle cell disease: definition, pathophysiology, and management. Eur J Haematol. 2020;105 (3):237–246. 10.1111/ejh.13430. 32301178

[68] McGill LS, Hamilton KR, Letzen JE, Depressive and insomnia symptoms sequentially mediate the association between racism-based discrimination in healthcare settings and clinical pain among adults with sickle cell disease. J Pain. 2022. 10.1016/j.jpain.2022.11.004. PMC10079566 36414154

[69] Smith WR, Scherer M. Sickle-cell pain: advances in epidemiology and etiology. Hematol 2010 Am Soc Hematol Educ Program Book. 2010;2010(1):409–415. 10.1182/asheducation-2010.1.409. 21239827

[70] Molokie RE, Wang ZJ, Yao Y, Sensitivities to thermal and mechanical stimuli: adults with sickle cell disease compared to healthy, pain-free african american controls. J Pain. 2020;21(9–10):957–967. 10.1016/j.jpain.2019.11.002. 31733363 PMC7217752

[71] Collins PJ, Renedo A, Marston CA. Communicating and understanding pain: limitations of pain scales for patients with sickle cell disorder and other painful conditions. J Health Psychol. 2022;27(1):103–118. 10.1177/1359105320944987. 32744117 PMC8739581

[72] Jin J, Yarns BC. The impact of stressful life events on centralized pain and pain intensity: a combined model examining the mediating roles of anger and perceived injustice among racially minoritized adults with chronic pain. J Pain. 2024;25(11), 104642. 10.1016/j.jpain.2024.104642. 39067581

[73] Saba SK, Davis JP, Prindle JJ, Bidirectional associations between pain and perceived stress among veterans: depressive disorder as a predisposing factor. Psychosom Med. 2024;86(1):44–51. 10.1097/PSY.0000000000001253. 37774110 PMC10841244

[74] White RS, Jiang J, Hall CB, Higher perceived stress scale scores are associated with higher pain intensity and pain interference levels in older adults. J Am Geriatr Soc. 2014;62(12):2350–2356. 10.1111/jgs.13135. 25516031 PMC4362541

[75] King CD, Keil A, Sibille KT Chapter 52 - Chronic Pain and Perceived Stress. In: Fink G, ed. Stress: Concepts, Cognition, Emotion, and Behavior. Academic Press; 2016:413–421.

[76] Atkinson TM, Liou KT, Borten MA, Association between music therapy techniques and patient-reported moderate to severe fatigue in hospitalized adults with cancer. JCO Oncol Pr. 2020;16(12):e1553–e1557. 10.1200/OP.20.00096. PMC7735038 32639926

[77] Werner LM, Skouras S, Bechtold L, Pallesen S, Koelsch S. Sensorimotor synchronization to music reduces pain. PLoS One. 2023;18(7), e0289302. 10.1371/journal.pone.0289302. 37506059 PMC10381080

[78] Howlin C, Stapleton A, Rooney B. Tune out pain: agency and active engagement predict decreases in pain intensity after music listening. PLoS One. 2022;17(8), e0271329. 10.1371/journal.pone.0271329. 35921262 PMC9348657

[79] Rodgers-Melnick SN, Lin L, Gam K, Effects of music therapy on quality of life in adults with sickle cell disease (MUSIQOLS): a mixed methods feasibility study. J Pain Res. 2022;15:71–91. 10.2147/JPR.S337390. 35046718 PMC8760983

[80] Koelsch S Brain correlates of music-evoked emotions. Nat Rev Neurosci. 2014;15(3):170–180. 10.1038/nrn3666. 24552785

[81] Melzack R. Pain and the neuromatrix in the brain. J Dent Educ. 2001;65(12):1378–1382. 11780656

[82] Sanfi I, Christensen E. Perspectives on music imagery and complex chronic pain. Approaches Music Ther Spec Educ. 2017;9:233–245.

[83] Klinger E, Carlini S, Gonzalez I, Accuracy of race, ethnicity, and language preference in an electronic health record. J Gen Intern Med. 2015;30(6):719–723. 10.1007/s11606-014-3102-8. 25527336 PMC4441665

